# Head-to-head comparison of glucose-to-albumin ratio against five albumin-derived indices for predicting 28-day ICU mortality in critically ill patients with spontaneous intracerebral hemorrhage: a multicenter retrospective study

**DOI:** 10.3389/fnut.2026.1909009

**Published:** 2026-10-08

**Authors:** Fangbin Hao, Luokai Huangfu, Wenxuan Li, Yanteng Li, Rui Hui, Caihong Sun, Meijing Liu, La Shi, Shenghan Xu, Gang Cheng, Jianning Zhang

**Affiliations:** 1 Senior Department of Neurosurgery, Chinese PLA General Hospital, Beijing, China; 2 Department of Neurosurgery, Affiliated Hospital of Guizhou Medical University, Guiyang, China; 3 Liupanshui People’s Hospital, Liupanshui, Guizhou, China; 4 The Affiliated Hospital of Changchun University of Traditional Chinese Medicine, Changchun, Jilin, China

**Keywords:** deep learning, glucose-to-albumin ratio, ICU mortality, intracerebral hemorrhage, risk stratification

## Abstract

**Background:**

Spontaneous intracerebral hemorrhage (ICH) carries a 30-day mortality exceeding 40%. The glucose-to-albumin ratio (GAR) reflects metabolic stress and nutritional status. Although previous studies have demonstrated its prognostic value in ICH, its utility specifically in ICU-admitted patients and its comparative performance against other albumin-derived indices remain incompletely characterized.

**Methods:**

We included 1,625 ICU ICH patients from MIMIC-IV (derivation) and 861 from two Chinese centers (external validation). Log-transformed GAR (LogGAR) was the exposure; 28-day ICU mortality was the primary outcome. Cox regression, restricted cubic splines, Kaplan–Meier, and Fine–Gray competing-risk models assessed LogGAR’s independent prognostic value, compared head-to-head with five albumin-derived indices. Lasso-selected features informed a multilayer perceptron (MLP), benchmarked against severity scores and a penalized logistic regression on identical features.

**Results:**

LogGAR was independently associated with 28-day ICU mortality (derivation: HR = 1.81, 95% CI 1.33–2.47, *p* < 0.001; external: HR = 3.48, 95% CI 2.54–4.78, *p* < 0.001), with linear dose–response (RCS P-nonlinear = 0.315). Fine–Gray competing-risk analysis confirmed robustness (subdistribution HR = 2.01, 95% CI 1.58–2.57, *p* < 0.001). Adding LogGAR to six severity scores improved discrimination (external ΔAUC = 0.085–0.107, all *p* < 0.001). Among six indices, LogGAR was comparable to logLDAR in the derivation cohort (0.679 vs. 0.681, *p* = 0.78) and highest in the external cohort (0.691). Subgroup analyses identified significant effect modification only for AKI (FDR = 0.029). The Lasso-MLP (8 features) and penalized logistic regression achieved comparable performance (validation AUC 0.718 for both; external AUC 0.759 vs. 0.783), indicating no added value from nonlinearity. Calibration slopes were 0.66 (validation) and 0.91 (external). SHAP identified LogGAR as the top predictor.

**Conclusion:**

LogGAR is independently associated with 28-day ICU mortality in critically ill patients with ICH and shows discriminative performance comparable to logLDAR and superior to the remaining four indices. A penalized logistic regression on Lasso-selected features matched the MLP, suggesting the predictive signal lies in features rather than model complexity—a pragmatic consideration for clinical implementation. As a readily available biomarker, LogGAR may support early ICU risk stratification.

## Introduction

Spontaneous intracerebral hemorrhage (ICH) is a critical disease caused by rupture of cerebral vessels, with high mortality and disability rates that impose heavy burdens on families and healthcare systems (1–3). Early identification of high-risk patients, timely intervention, and accurate prognostication are essential to reduce mortality and manage complications (4, 5). Although several clinical scoring systems are available, their complexity limits widespread use (6–8). Recently, the role of nutritional status in acute ICH has gained attention, as metabolic and nutritional parameters are prognostic yet largely absent from existing scores (9, 10). Thus, identifying easily accessible biomarkers reflecting both nutritional imbalance and metabolic stress has become a research priority.

The glucose-to-albumin ratio (GAR) integrates blood glucose and albumin into a single index, reflecting both metabolic stress and protein nutritional status (9–11). Compared with single indicators, GAR simultaneously addresses two modifiable targets: hyperglycemia and hypoalbuminemia. In acute ICH, stress hormones promote hepatic glucose output and peripheral insulin resistance, leading to stress-induced hyperglycemia (SIH), which exacerbates neuronal injury via oxidative stress and inflammation (12, 13). Concurrently, albumin (ALB)—which maintains colloid osmotic pressure and exerts anti-inflammatory and antioxidant effects (14, 15)—is often depleted in critically ill ICH patients due to rapid catabolism, weakening its protective functions and associating with poor outcomes. By capturing both pathways, GAR can help identify high-risk patients and guide nutritional interventions (e.g., glycemic control and protein supplementation) to potentially improve short-term outcomes.

Previous studies have established GAR as a promising prognostic biomarker in ICH (16). However, three knowledge gaps remain: (1) no study has systematically compared GAR with other albumin-derived composite indices (e.g., logLDAR, RAR, UAR, TAR, AGAR) in ICH; (2) its utility specifically for the 28-day ICU mortality endpoint—the key window for acute intervention—has not been characterized; and (3) the incremental value of deep learning over simple linear models in predicting ICH outcomes has not been explored. Therefore, this study aimed to systematically evaluate the independent predictive value of GAR in ICU-admitted ICH patients, using a large public database for derivation and a two-center cohort for external validation, while performing head-to-head comparisons, competing-risk analysis, and linear versus deep learning model comparisons to determine whether nonlinearity adds predictive value.

## Methods

### Data sources

This retrospective study used two cohorts. The derivation cohort was from the MIMIC-IV database (version 2.2) (17), containing 196,527 adult patients admitted to Beth Israel Deaconess Medical Center (2008–2019). The MIT Institutional Review Board approved the use of MIMIC-IV and waived informed consent due to the retrospective, de-identified nature of the data. The external validation cohort comprised 861 ICH patients from two centers: Chinese PLA General Hospital (n = 321) and Affiliated Hospital of Guizhou Medical University (n = 540), enrolled between January 2025 and June 2026. The study was approved by the Ethics Committee of the Affiliated Hospital of Guizhou Medical University (approval No. 20261018) and the IRB of the Chinese PLA General Hospital (approval No. S2025-751-01), with informed consent waived owing to the retrospective design. All external patients were ICU-admitted, ensuring consistent outcome definition (ICU mortality) across cohorts.

### Study population

Data were extracted from MIMIC-IV using PostgreSQL (v13.7.2) and Navicat Premium (v16). We included adult patients (age ≥ 18 years) with a diagnosis of ICH confirmed by ICD-9 code 431 and ICD-10 codes I610–I619 and I62.9 who were admitted to the ICU. Exclusion criteria were: (1) death within 24 h after ICU admission, to avoid incomplete data or negative survival times; (2) multiple ICU admissions, retaining only the first ICH-related record to prevent data duplication; and (3) absence of key laboratory parameters needed to calculate GAR at the first ICU stay. The patient selection process is illustrated in Supplementary Figure S1.

### Variable extraction and handling

Data extraction was performed using PostgreSQL (13.7.2), Navicat Premium (16.0), and SQL. Extracted variables were categorized into six domains: demographics, comorbidities, vital signs, laboratory tests, severity scores, and therapeutic interventions (Supplementary Table S1). The primary exposure was GAR, calculated as the ratio of the first serum glucose measurement to the serum albumin measurement obtained after ICU admission. Due to marked right-skewness, GAR was natural log-transformed (LogGAR) for all analyses. The primary outcome was 28-day ICU mortality.

Missing data were handled via multiple imputation using the mice package (v3.16.0, *m* = 10, maxit = 50, predictive mean matching) (18). Among 40 analysis variables, 21 were fully complete and 19 had missingness between 1.1% and 5.9% (maximum: INR); variables with >30% missingness were excluded. The imputation model included all analysis variables, LogGAR, and the outcome. Convergence was confirmed by trace plots (stable mean and SD, no drift) and Gelman–Rubin PSRF <1.05 for all imputed variables, confirming robustness. Missingness proportions are provided in Supplementary materials.

### Association between LogGAR and clinical outcomes

After verifying the proportional hazards assumption using Schoenfeld residual tests (all global *p* > 0.05), we used Cox proportional hazards regression (19) to assess the association between LogGAR and 28-day ICU mortality. Three progressively adjusted models were built: Model 1 included LogGAR alone; Model 2 additionally adjusted for demographics (age, sex, race, weight) and baseline comorbidities (acute kidney injury [AKI], chronic kidney disease [CKD], hyperlipidaemia [HLD], heart failure [HF], myocardial infarction [MI], chronic obstructive pulmonary disease [COPD]); Model 3 further added key laboratory indices and therapeutic measures (non-invasive systolic blood pressure [NBPS], non-invasive diastolic blood pressure [NBPD], non-invasive mean pressure [NBPM], oxygen saturation [SpO_2_], red blood cell count [RBC], white blood cell count [WBC], anion gap [AG], potassium [K], total carbon dioxide [CO_2_CP], lactate [Lac], pH, partial pressure of oxygen [PO_2_], partial thromboplastin time [PTT], alanine aminotransferase [ALT], total bilirubin [TB], creatinine [CREA], serum albumin [ALB], vasopressor use [VP], glucocorticoid use [GC], antihypertensive therapy [AHT], antibiotic therapy [ABX], sedation [SED], continuous renal replacement therapy [CRRT]) based on prior literature (20–22) and after excluding collinear factors (variance inflation factor [VIF] > 5). Restricted cubic spline [RCS] regression (23) with knots at the 5th, 35th, 65th, and 95th percentiles explored nonlinear relationships, and Kaplan–Meier curves visualized survival differences. Time zero was ICU admission; patients discharged alive before day 28 were censored at discharge, and those still in the ICU at day 28 were censored at day 28.

### Incremental effect of LogGAR

We added LogGAR to each of six conventional severity scores and built multivariate logistic regression models. ROC curves and AUC assessed predictive performance; the DeLong test (24) compared AUCs before and after adding LogGAR. For reclassification, we used risk categories of predicted mortality probability <10%, 10–20, and >20% to calculate the categorical net reclassification improvement (cNRI (25)) with bootstrap 95% CI (500 resamples). These thresholds align with common ICH mortality risk stratification and have been used in prior ICH studies (6–8). We also report continuous NRI and integrated discrimination improvement (IDI) as threshold-independent metrics.

### Subgroup and interaction analyses of LogGAR

To validate the robustness of the association between LogGAR and the clinical endpoint, we performed subgroup analyses and interaction tests according to a pre-specified analysis plan. Subgroups were defined based on important clinical characteristics, including age, sex, race and baseline comorbidities. By calculating the significance level of the interaction term (i.e., interaction *p* value), we assessed whether the effect of LogGAR was consistent across subgroups. If the interaction test for all subgroups was not statistically significant (usually defined as *p* > 0.05), this indicated that the association was stable across different populations; otherwise, it suggested heterogeneity of effect. All analyses strictly followed the pre-specified analysis procedure to control the risk of bias from multiple comparisons.

### Comparison of LogGAR with five other albumin-derived indices

We compared LogGAR with five albumin-derived indices: RAR (RDW/ALB), AGAR (AG/ALB), UAR (URE/ALB), TAR (TB/ALB), and logLDAR [log(LDH/ALB)]. Comparisons were performed across four dimensions: (1) AUC with bootstrap 95% CI and pairwise DeLong tests with Benjamini–Hochberg correction across 15 comparisons; (2) optimal Youden cut-off derived from the derivation cohort, with diagnostic performance evaluated at that threshold in both cohorts; (3) univariate and multivariate Cox regression for each index, excluding its component variables (e.g., Glu and ALB for LogGAR) and confirmed by full correlation (*r* = 0.000) between each index and its computed ratio; and (4) mean cNRI across the six severity scores (36 combinations) as a summary measure for each index.

### Deep learning prediction model

Lasso regression (L1 regularization) with 10-fold cross-validation was applied to 28 candidate features (22 laboratory variables with univariate associations with ICU mortality, *p* value <0.05; plus 6 albumin-derived indices) (26). The optimal *λ* minimized cross-validated mean squared error, retaining features with non-zero coefficients; the L1 penalty naturally handled collinearity.

A multilayer perceptron with three hidden layers (128, 64, 32 neurons), batch normalization, ReLU activation, and dropout (0.3) was trained using weighted binary cross-entropy loss (pos_weight = 4.39) for class imbalance (event rate 18.5%) (27). Optimization used Adam (lr = 0.001, weight_decay = 1e-4) with ReduceLROnPlateau and early stopping (patience = 20). The derivation cohort was randomly split into training (70%, *n* = 1,137) and validation (30%, *n* = 488) sets; features were standardized using training-set statistics. Model performance was evaluated on the training, validation, and external cohorts (*n* = 861), with full-derivation performance omitted to avoid circular evaluation. Metrics included AUC, sensitivity, specificity, PPV, NPV, F1, Youden index, Brier score, and AUPRC; feature importance was assessed using SHAP with Kernel Explainer (28).

A penalized logistic regression (L2 ridge, C = 1.0, class-weight balanced) was trained on the identical eight Lasso-selected features using the same split and standardization to test whether MLP nonlinearity added value over a linear model. The six conventional severity scores (SOFA, APSIII, SAPSII, OASIS, Charlson, APACHEII) were used as fixed published instruments and not refit on our data; this asymmetry is acknowledged in the Discussion. Calibration was evaluated using calibration plots with slope and intercept. Reporting follows TRIPOD, with TRIPOD-AI considerations applied for the machine-learning component.

### Sensitivity analyses

To assess robustness, three prespecified sensitivity analyses were performed. First, to address potential misclassification of ICD-10 code I62.9 (which may include non-parenchymal haemorrhages), we excluded all such cases and re-estimated the association between LogGAR and mortality using multivariable logistic regression with the same covariates as in the primary Model 3. Second, to account for unadjusted neurological severity, we incorporated the GCS score into Model 3 within the internal cohort. Third, to account for informative censoring due to ICU discharge before day 28, we performed a Fine-Gray competing risk analysis (using the cmprsk package), treating alive ICU discharge as a competing event, and estimated subdistribution hazard ratios (sHR) with and without covariate adjustment. All results were compared with the primary Cox regression findings.

### Statistical analysis

LogGAR was divided into tertiles (low, middle, high). Continuous variables were described as mean ± SD or median (IQR) and compared using parametric or nonparametric tests as appropriate; categorical variables were presented as counts (percentages) and compared using chi-squared or Fisher’s exact tests. All tests were two-sided with *p* < 0.05 considered significant. For subgroup interaction analyses, Benjamini-Hochberg FDR correction was applied across all interaction *p*-values; interactions with FDR < 0.05 were considered significant, while others were labeled exploratory. Analyses were conducted using R version 4.5.1.

## Results

### Baseline characteristics of the study population

A total of 1,625 critically ill ICH patients were included (median age 67.0 years, IQR 54.0–78.0; 59.9% male). Baseline characteristics stratified by LogGAR tertiles are shown in Table 1. Compared with the low LogGAR group, patients in the high LogGAR group had significantly higher proportions of AKI, DM, IHD, and CKD (all *p* < 0.01), greater use of mechanical ventilation, CRRT, sedation, vasopressors, and glucocorticoids (all *p* < 0.001), and more unstable vital signs (lower NBPS/NBPM; higher HR and RR). Laboratory findings in the high LogGAR group showed elevated RDW, K, AG, INR, PTT, PT, CREA, and URE, with decreased ALB, CO_2_CP, and pH (all *p* < 0.05), indicating more pronounced metabolic disturbance. All clinical severity scores (SOFA, APSIII, SAPSII, OASIS, Charlson, APACHE II) increased with LogGAR tertiles (all *p* < 0.001). The high LogGAR group also had higher ICU mortality (29.7% vs. 8.1%, *p* < 0.001) and longer ICU stay (median 7.51 vs. 4.11 days, *p* < 0.001).

**Table 1:** Baseline Characteristics of ICH Patients by GAR Tertiles.

Variable	Total (*N* = 1,625)	Low (*N* = 541)	Moderate (*N* = 541)	High (*N* = 543)	*p*-value
Age	67.00 (54.00–78.00)	65.00 (52.00–77.00)	68.00 (53.00–79.00)	67.00 (57.00–78.00)	0.077
Gender	Male: 973 (59.9%)	Male: 325 (60.1%)	Male: 331 (61.2%)	Male: 317 (58.4%)	0.638
Race	White: 882 (54.3%)	White: 311 (57.5%)	White: 292 (54.0%)	White: 279 (51.4%)	0.129
Weight	75.50 (64.30–90.00)	73.50 (62.80–86.40)	74.53 (63.90–87.00)	80.00 (66.80–95.65)	<0.001
HTN	835 (51.4%)	283 (52.3%)	294 (54.3%)	258 (47.5%)	0.069
AKI	442 (27.2%)	105 (19.4%)	127 (23.5%)	210 (38.7%)	<0.001
CKD	213 (13.1%)	60 (11.1%)	60 (11.1%)	93 (17.1%)	0.003
DM	379 (23.3%)	52 (9.6%)	96 (17.7%)	231 (42.5%)	<0.001
HLD	507 (31.2%)	152 (28.1%)	173 (32.0%)	182 (33.5%)	0.140
IHD	357 (22.0%)	94 (17.4%)	105 (19.4%)	158 (29.1%)	<0.001
COPD	150 (9.2%)	45 (8.3%)	58 (10.7%)	47 (8.7%)	0.335
SOFA	4.00 (2.00–6.00)	3.00 (2.00–5.00)	4.00 (2.00–5.00)	5.00 (3.00–7.00)	<0.001
APSIII	39.00 (29.00–51.00)	34.00 (25.00–44.00)	39.00 (29.00–48.00)	47.00 (34.00–59.50)	<0.001
SAPSII	34.00 (27.00–42.00)	30.00 (24.00–38.00)	34.00 (27.00–41.00)	38.00 (30.00–48.00)	<0.001
OASIS	32.00 (27.00–37.00)	30.00 (25.00–35.00)	32.00 (28.00–37.00)	34.00 (29.50–40.00)	<0.001
Charlson	5.00 (3.00–7.00)	5.00 (3.00–7.00)	5.00 (3.00–7.00)	6.00 (4.00–8.00)	<0.001
APACHEII	15.00 (11.00–20.00)	13.00 (10.00–18.00)	15.00 (11.00–20.00)	17.00 (13.00–23.00)	<0.001
HR	85.00 (72.00–98.00)	82.00 (70.00–93.00)	84.00 (72.00–97.00)	90.00 (77.00–104.00)	<0.001
NBPS	130.00 (116.00–147.00)	132.00 (119.00–147.00)	131.00 (118.00–147.00)	127.00 (110.00–147.00)	0.023
NBPD	73.00 (62.00–85.00)	76.00 (65.00–87.00)	72.00 (61.00–85.00)	70.00 (60.00–83.00)	<0.001
NBPM	88.00 (78.00–100.00)	91.00 (80.00–102.00)	87.00 (78.00–99.00)	85.00 (74.00–99.00)	<0.001
RR	18.00 (16.00–22.00)	18.00 (15.00–21.00)	18.00 (15.00–22.00)	19.00 (16.00–23.00)	<0.001
SpO2	98.00 (96.00–100.00)	98.00 (96.00–100.00)	99.00 (96.00–100.00)	99.00 (96.00–100.00)	0.229
HCT	35.30 (30.80–39.10)	36.30 (32.30–40.00)	35.00 (31.20–39.10)	34.00 (28.95–38.30)	<0.001
Hb	11.70 (10.10–13.10)	12.10 (10.70–13.30)	11.70 (10.20–12.90)	11.30 (9.60–12.90)	<0.001
PLT	194.00 (142.00–255.00)	190.00 (146.00–249.00)	203.00 (144.00–253.00)	192.00 (131.00–259.00)	0.236
RDW	13.90 (13.10–15.20)	13.70 (13.00–15.00)	14.00 (13.20–15.10)	14.10 (13.20–15.40)	0.005
RBC	3.86 (3.32–4.35)	4.04 (3.48–4.46)	3.86 (3.37–4.30)	3.71 (3.17–4.25)	<0.001
WBC	10.70 (8.00–14.40)	9.20 (7.00–12.40)	10.40 (8.10–14.00)	12.70 (9.40–16.95)	<0.001
ALB	3.40 (3.00–3.80)	3.70 (3.40–4.00)	3.30 (3.00–3.70)	3.00 (2.60–3.40)	<0.001
AG	14.00 (12.00–17.00)	14.00 (12.00–17.00)	14.00 (12.00–16.00)	15.00 (13.00–17.00)	0.005
Ca	8.60 (8.10–9.00)	8.80 (8.30–9.10)	8.50 (8.00–9.00)	8.40 (7.90–8.90)	<0.001
Cl	104.00 (100.00–107.00)	103.00 (100.00–106.00)	104.00 (101.00–107.00)	104.00 (100.00–108.00)	0.043
Glu	131.00 (108.00–167.00)	103.00 (92.00–114.00)	131.00 (117.00–148.00)	187.00 (158.00–228.50)	<0.001
K	4.00 (3.60–4.40)	4.00 (3.60–4.30)	3.90 (3.60–4.30)	4.00 (3.70–4.50)	<0.001
Na	139.00 (137.00–142.00)	140.00 (137.00–142.00)	139.00 (137.00–142.00)	139.00 (136.00–141.00)	0.004
CO_2_CP	24.00 (22.00–27.00)	25.00 (23.00–27.00)	25.00 (22.00–27.00)	24.00 (21.00–26.00)	<0.001
PCO_2_	39.00 (33.00–45.00)	39.00 (33.00–45.00)	38.00 (33.00–44.00)	39.00 (33.00–46.00)	0.185
PH	7.40 (7.34–7.45)	7.41 (7.35–7.45)	7.41 (7.36–7.46)	7.39 (7.32–7.44)	<0.001
PO_2_	116.00 (73.00–190.00)	112.00 (72.00–177.00)	116.00 (77.00–199.00)	118.00 (74.00–196.00)	0.223
INR	1.20 (1.10–1.30)	1.10 (1.10–1.30)	1.20 (1.10–1.30)	1.20 (1.10–1.40)	<0.001
PT	13.00 (11.90–14.70)	12.60 (11.60–13.90)	13.00 (12.00–14.70)	13.40 (12.20–15.30)	<0.001
PTT	28.50 (25.80–31.90)	28.60 (26.10–31.50)	28.10 (25.30–31.40)	29.00 (26.00–32.85)	0.013
ALT	24.00 (16.00–44.00)	21.00 (14.00–36.00)	24.00 (16.00–42.00)	28.00 (18.00–58.00)	<0.001
AST	33.00 (21.00–60.00)	28.00 (19.00–48.00)	33.00 (22.00–58.00)	41.00 (25.00–74.00)	<0.001
TB	0.60 (0.40–1.00)	0.60 (0.40–0.90)	0.60 (0.40–0.90)	0.60 (0.40–1.00)	0.962
CRE	0.90 (0.70–1.20)	0.80 (0.70–1.10)	0.90 (0.70–1.10)	1.00 (0.80–1.40)	<0.001
URE	16.00 (12.00–24.00)	14.00 (11.00–21.00)	16.00 (12.00–22.00)	19.00 (14.00–30.00)	<0.001
LDH	266.00 (209.00–365.00)	237.00 (191.00–321.00)	264.00 (210.00–359.00)	296.00 (230.00–405.50)	<0.001
Mg	1.90 (1.70–2.10)	1.90 (1.80–2.10)	1.90 (1.70–2.10)	1.90 (1.60–2.10)	0.002
Ventilation	1,290 (79.4%)	370 (68.4%)	448 (82.8%)	472 (86.9%)	<0.001
CRRT	72 (4.4%)	10 (1.8%)	21 (3.9%)	41 (7.6%)	<0.001
SA	1,090 (67.1%)	282 (52.1%)	370 (68.4%)	438 (80.7%)	<0.001
VP	690 (42.5%)	126 (23.3%)	228 (42.1%)	336 (61.9%)	<0.001
GC	411 (25.3%)	107 (19.8%)	154 (28.5%)	150 (27.6%)	0.001
AHT	1,117 (68.7%)	299 (55.3%)	392 (72.5%)	426 (78.5%)	<0.001
ABX	1,257 (77.4%)	342 (63.2%)	430 (79.5%)	485 (89.3%)	<0.001
ICU time	5.52 (2.65–12.13)	4.11 (2.18–7.91)	6.10 (2.81–13.05)	7.51 (3.21–15.59)	<0.001
ICU dead	301 (18.5%)	44 (8.1%)	96 (17.7%)	161 (29.7%)	<0.001

### Association between LogGAR and 28-day ICU mortality

Cox regression analysis showed a significant association between LogGAR and the risk of death (Table 2). In the unadjusted Model 1, LogGAR was strongly associated with mortality (HR = 2.20, 95% CI: 1.72–2.82, *p* < 0.001). This association remained strong after adjusting for demographics and comorbidities in Model 2 (HR = 1.97, 95% CI: 1.50–2.58, *p* < 0.001). In the fully adjusted Model 3, LogGAR retained independent prognostic significance (HR = 1.81, 95% CI: 1.33–2.47, *p* < 0.001). When LogGAR was analyzed by three groups, compared with the low GAR group (reference), the HR for the middle group in the fully adjusted model was 1.73 (95% CI: 1.19–2.53, *p* = 0.004) and for the high group was 2.05 (95% CI = 1.39–3.01, *p* < 0.001). These findings indicate that LogGAR has independent and graded prognostic value for ICU mortality, with a sustained effect after comprehensive adjustment. Similarly, the RCS curve further revealed a linear positive dose–response relationship between LogGAR and short-term mortality in critically ill ICH patients (Figure 1A). Subsequent Kaplan–Meier survival curves showed that the high LogGAR group, split by the median, had shorter survival times compared with the low LogGAR group (Figure 1C).

**Table 2:** Cox regression results of LogGAR and 28 day ICU mortality rate in critically ill ICH patients.

Characteristic	Model 1			Model 2			Model 3		
	HR	95% CI	*p*-value	HR	95% CI	*p*-value	HR	95% CI	*p*-value
LogGAR	2.20	1.72, 2.82	<0.001	1.97	1.50, 2.58	<0.001	1.81	1.33, 2.47	<0.001
LogGAR group									
Low	Ref	Ref		Ref	Ref		Ref	Ref	
Moderate	1.52	1.06, 2.17	0.023	1.50	1.04, 2.14	0.028	1.73	1.19, 2.53	0.004
High	2.25	1.61, 3.15	<0.001	2.04	1.44, 2.89	<0.001	2.05	1.39, 3.01	<0.001

CI, confidence interval, HR, hazard ratio.

**Figure 1:**
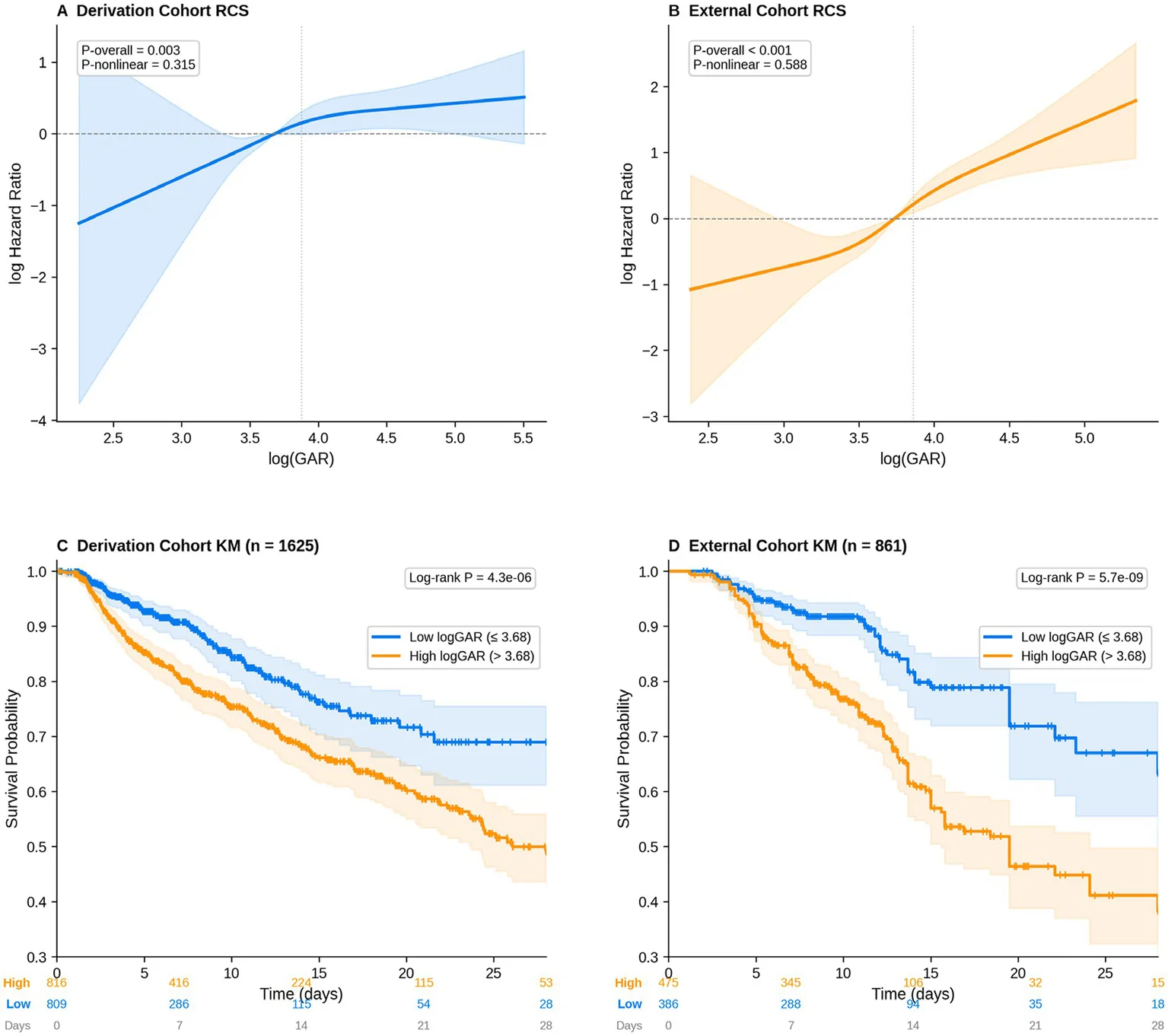
Association between LogGAR and 28-day ICU mortality in the derivation and external validation cohorts. **(A)** RCS curve in the derivation cohort (*n* = 1,625, 301 events), adjusted for Age, AKI, SOFA (P-overall = 0.003, P-nonlinear = 0.315). **(B)** RCS curve in the external cohort (*n* = 861, 209 events), adjusted for Age, AKI, SOFA, GCS (P-overall < 0.001, P-nonlinear = 0.588). **(C)** KM curves in the derivation cohort stratified by median LogGAR (cutoff = 3.68; log-rank *p* < 0.001; HR = 2.20, 95% CI: 1.72–2.82). **(D)** CIF curves from the Fine-Gray competing-risk model in the external cohort, showing consistently higher cumulative mortality in the high LogGAR group.

### Incremental predictive value of LogGAR

When LogGAR was added to each of the six conventional severity scores, AUC consistently improved: SOFA increased from 0.699 to 0.748 (ΔAUC = +0.049), APS III from 0.699 to 0.738 (+0.039), SAPS II from 0.699 to 0.736 (+0.037), OASIS from 0.680 to 0.721 (+0.042), Charlson from 0.578 to 0.692 (+0.114), and APACHE II from 0.697 to 0.739 (+0.042), with DeLong test *p* values all < 0.05 (Figure 2). cNRI confirmed significant improvements in risk reclassification for all six scores: SOFA (cNRI = 0.241, *p* < 0.001), APS III (0.161, *p* < 0.001), SAPS II (0.165, *p* < 0.001), OASIS (0.137, *p* < 0.001), Charlson (0.266, *p* < 0.001) and APACHE II (0.121, *p* = 0.004). Reclassification benefit was mainly driven by the non-event component (correct downward reclassification of survivors), although the event component also contributed positively (Figure 2). The continuous NRI (without risk categories) and IDI further confirmed significant improvements: continuous NRI ranged from 0.343 (SOFA) to 0.443 (Charlson), and IDI ranged from 0.025 (SOFA) to 0.052 (Charlson), all indicating meaningful improvement in predicted probabilities when LogGAR was added. These results indicate that LogGAR provides incremental prognostic information beyond established clinical severity scores, improving discrimination and risk reclassification.

**Figure 2:**
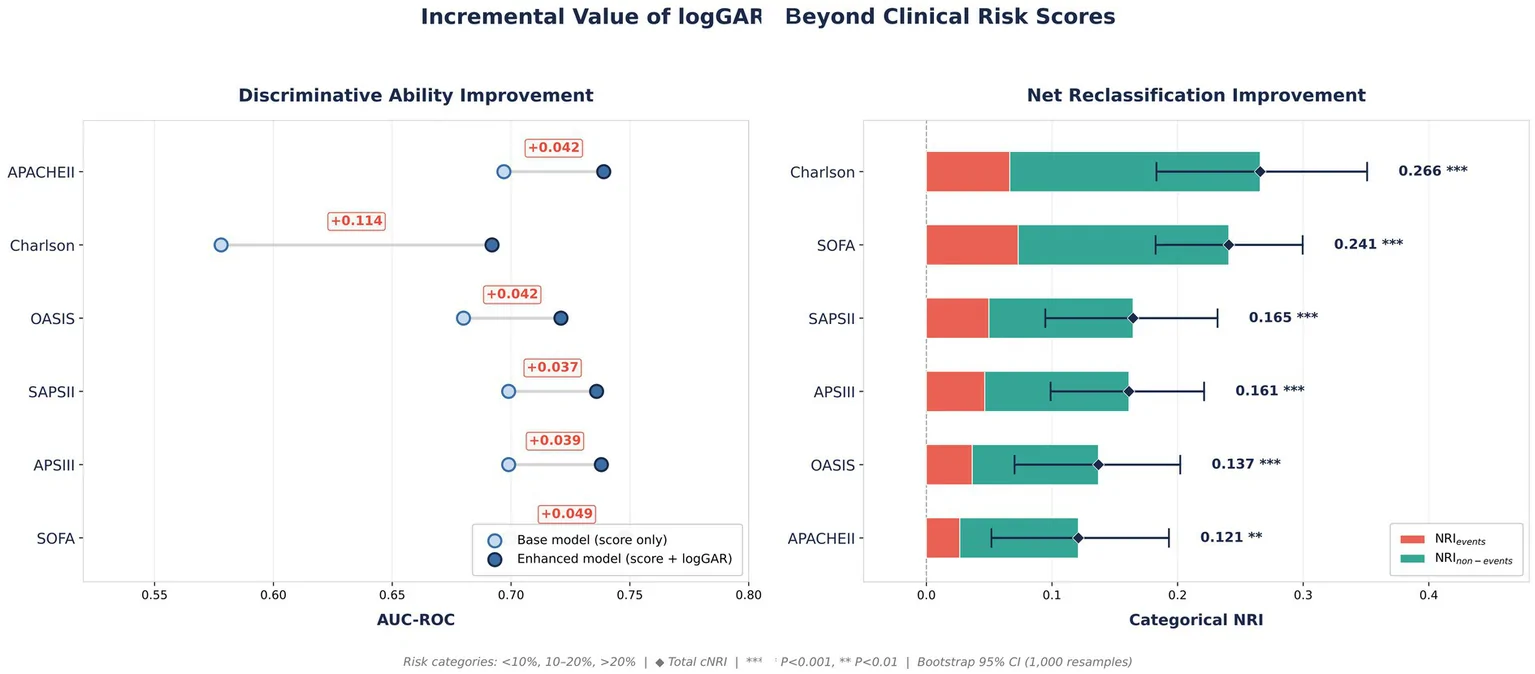
Incremental predictive value of adding LogGAR to six severity scores (derivation cohort). Bars show AUC for each score alone (light grey) and with LogGAR (dark grey). ΔAUC (above each pair) and categorical NRI (cNRI, value above bar) are shown. All ΔAUC were significant (DeLong test, *p* < 0.05). cNRI calculated using three risk categories (<10%, 10–20, >20%) with bootstrap 95% CI. cNRI *p*-values: SOFA <0.001, APS III < 0.001, SAPS II < 0.001, OASIS <0.001, Charlson <0.001, APACHE II = 0.004.

### Subgroup and interaction analyses of LogGAR

To evaluate the consistency of the association between LogGAR and mortality across different clinical subgroups, we performed subgroup analyses and interaction analyses (Figure 3). After Benjamini–Hochberg correction for multiplicity across all interaction tests, only the AKI interaction remained significant (interaction *p* = 0.003, BH-FDR = 0.029). The diabetes (*p* = 0.027, BH-FDR = 0.134) and COPD (*p* = 0.043, BH-FDR = 0.142) interactions did not survive multiple-comparison correction and are therefore considered exploratory findings requiring validation. In most subgroups, including age, race, HTN, CKD, HLD and IHD, the association between LogGAR and 28-day mortality remained stable (interaction *p* > 0.05). The effect of LogGAR was very strong in patients without AKI (HR = 3.12) but attenuated in patients with AKI (HR = 1.39, *p* = 0.077).

**Figure 3:**
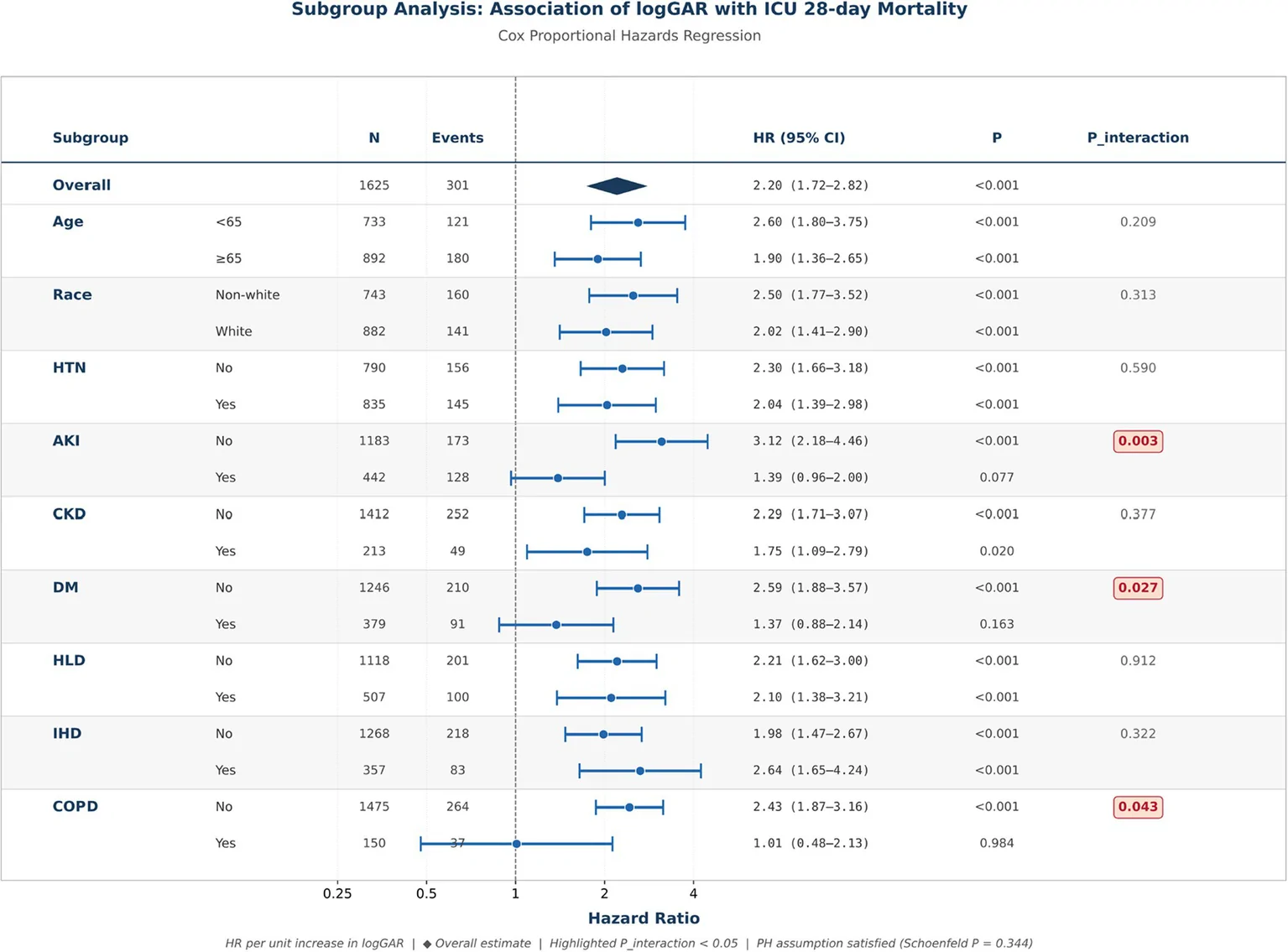
Subgroup and interaction analyses for LogGAR and 28-day ICU mortality. Forest plot showing HRs for LogGAR (per unit increase) within each subgroup, adjusted for Model 3 covariates except the stratification variable. Square size proportional to subgroup sample size; horizontal lines represent 95% CI. Interaction *p*-values with BH-FDR correction shown on the right. Only AKI survived FDR correction (FDR = 0.029); diabetes (FDR = 0.134) and COPD (FDR = 0.142) were exploratory.

### Comparison of predictive performance among albumin-derived indices

First, in multivariate Cox regression analysis, LogGAR (HR = 1.81, *p* < 0.001), logLDAR (HR = 1.588, *p* < 0.001), TAR (HR = 1.25, *p* < 0.001), RAR (HR = 1.09, *p* = 0.034) and UAR (HR = 1.02, *p* = 0.026) all had independent prognostic significance in critically ill ICH patients, while AGAR did not reach statistical significance (HR = 1.04, *p* = 0.334). ROC curve analysis (Figure 4A) showed that logLDAR and LogGAR had similar predictive performance for poor prognosis in critically ill ICH patients, with AUCs of 0.681 and 0.679, respectively (DeLong test *p* > 0.05), and both were higher than RAR (0.659), UAR (0.653), TAR (0.633) and AGAR (0.631), with paired DeLong test *p* values all < 0.05. At the optimal cut-off determined by the Youden index, LogGAR exhibited the highest sensitivity (77.7%) and negative predictive value (NPV, 91.0%) among all indices, with a cut-off of 3.638, specificity of 51.2% and Youden index of 0.289. In terms of incremental predictive value for the six severity scores, the ranking of the mean categorical net reclassification index (cNRI) was: LogGAR highest (0.182), followed by logLDAR (0.162), RAR (0.096), AGAR (0.064), UAR (0.050) and TAR (0.037) (Figure 4C). These results indicate that among the six indices, LogGAR and logLDAR had the strongest discriminative ability and prognostic value, with LogGAR performing better in terms of sensitivity, independent prognostic significance and incremental reclassification improvement.

**Figure 4:**
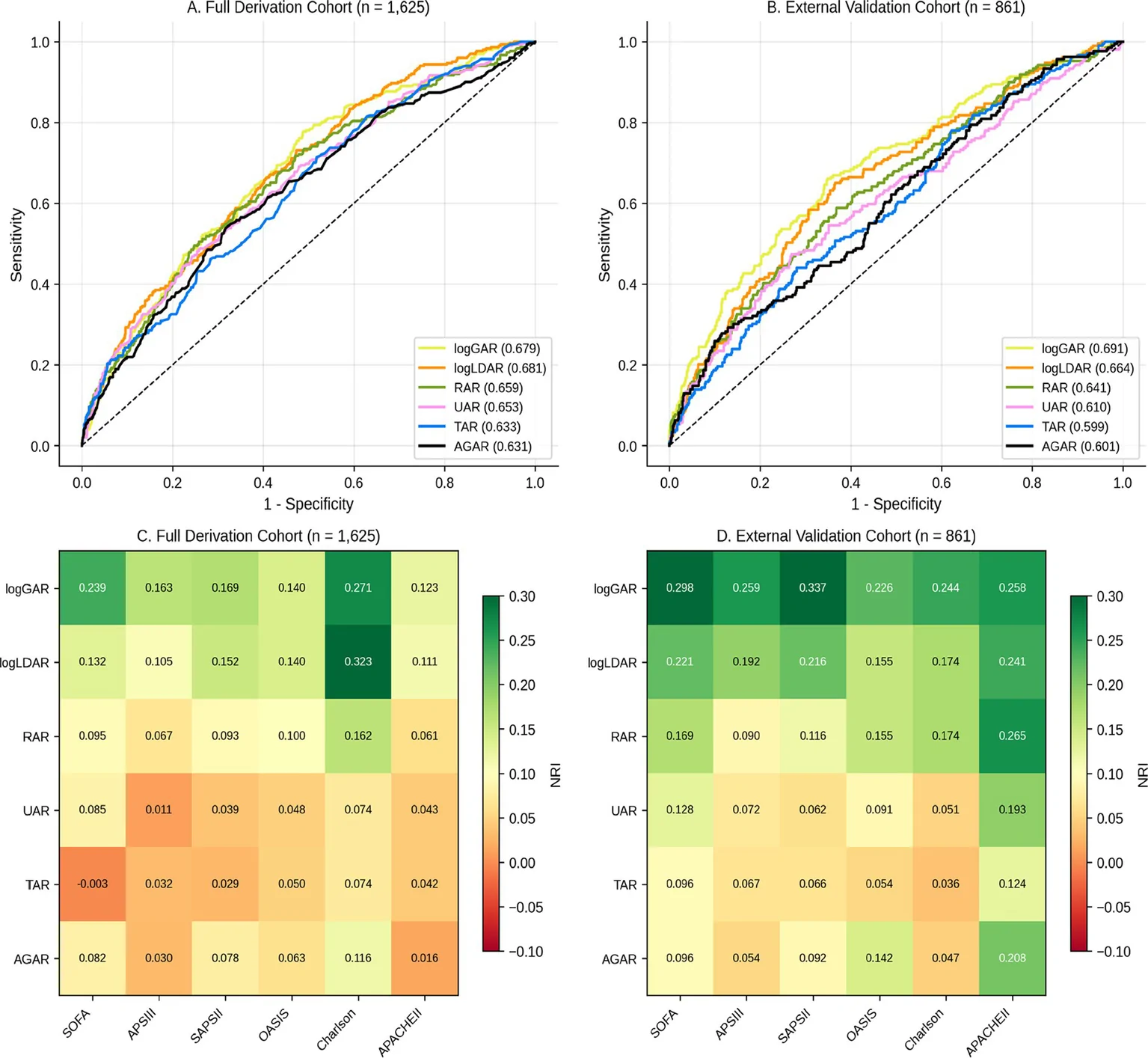
Head-to-head comparison of six albumin-derived indices (LogGAR, logLDAR, RAR, UAR, TAR, AGAR) for predicting 28-day ICU mortality. **(A)** ROC curves in derivation cohort (*n* = 1,625). **(B)** ROC curves in external cohort (n = 861); LogGAR showed the highest AUC (0.691). **(C)** cNRI matrix in derivation cohort. **(D)** cNRI matrix in external cohort. Risk categories: <10%, 10–20, >20%.

### External validation of LogGAR in an independent cohort

In the external validation cohort (N = 861, 28-day ICU mortality 24.3%; Supplementary Figure S3) baseline characteristics of the external cohort are shown in Supplementary Table S2, and a cross-cohort comparison is presented in Supplementary Table S3. LogGAR remained independently associated with mortality across sequential adjustments: unadjusted HR = 3.72 (95% CI 2.71–5.12, *p* < 0.001); demographics+comorbidities HR = 3.63 (95% CI 2.64–4.99, *p* < 0.001); parsimonious Model 3 (LogGAR, Age, AKI, SOFA, GCS; EPV = 41.8) HR = 3.48 (95% CI 2.54–4.78, *p* < 0.001). After additional adjustment for hematoma volume, location, IVH, expansion, and neurosurgical intervention (EPV = 20.9), LogGAR remained strongly associated with mortality (HR = 3.77, 95% CI 2.74–5.18, *p* < 0.001). Among the six indices, LogGAR showed the highest AUC (0.691, 95% CI 0.649–0.733) and AUPRC (0.452), outperforming logLDAR (AUC 0.664), RAR (0.641), UAR (0.610), TAR (0.599), and AGAR (0.601) (Figure 4B). Adding LogGAR to six severity scores significantly improved discrimination (ΔAUC = 0.085–0.107, all *p* < 0.001) (Figure 4D). Restricted cubic spline analysis confirmed a significant linear dose–response (P-overall<0.001, P-nonlinear = 0.588; 3-knot and 5-knot sensitivity analyses *p* = 0.907 and 0.781, respectively), and Kaplan–Meier analysis showed a significant survival difference between high and low LogGAR groups (HR = 2.44, 95% CI 1.79–3.32, log-rank *p* = 5.7 × 10^−9^) (Figures 1B,D). Collectively, these findings consistently support the prognostic stability of LogGAR across different adjustment models, analytical approaches, and comparison with other albumin-derived indices in an independent external cohort.

### Sensitivity analyses

To assess robustness, three prespecified sensitivity analyses were performed. First, excluding ICD-10 I62.9 cases (*n* = 47 removed) to address potential misclassification, LogGAR remained significantly associated with 28-day ICU mortality (OR = 1.80, 95% CI 1.32–2.46, *p* < 0.001), virtually unchanged from the primary analysis (HR = 1.81). Second, incorporating GCS into the fully adjusted Model 3 yielded a nearly identical HR of 1.79 (95% CI 1.31–2.44, *p* < 0.001), confirming robustness to neurological severity adjustment. Third, Fine–Gray competing-risk analysis treating alive ICU discharge before day 28 as a competing event (*n* = 1,244) showed that LogGAR remained strongly associated with the cumulative incidence of mortality (unadjusted sHR = 3.19, 95% CI 2.59–3.93, *p* < 0.001; parsimoniously adjusted sHR = 2.01, 95% CI 1.58–2.57, *p* < 0.001). Collectively, these analyses confirm that the independent prognostic value of LogGAR is robust to stricter diagnostic definitions, adjustment for neurological severity, and competing risks (Supplementary Figure S2).

### Deep learning prediction model

Lasso regression with 10-fold cross-validation (*λ* = 0.0113) selected eight features with non-zero coefficients from 28 candidates: LogGAR (0.073), logLDAR (0.029), RDW (0.025), PT (0.023), PTT (0.022), TB (0.021), URE (0.017), and AG (0.017) (Figure 5). To assess whether MLP nonlinearity added value over a linear model, a penalized logistic regression (L2 ridge) was trained on the identical eight features. The MLP and penalized LR achieved comparable AUCs (training curves in Supplementary Figure S4) (validation: 0.718 for both; external: 0.759 vs. 0.783), and the two models were statistically indistinguishable on the validation set (DeLong *p* = 0.948), with the penalized LR numerically superior externally, indicating that nonlinearity did not extract additional signal from these predictors. Calibration slopes for the MLP were 0.66 (validation) and 0.91 (external) (Figure 6), suggesting overconfidence/overfitting in the smaller validation sample but improved calibration in the external cohort. SHAP analysis identified LogGAR as the top predictor (mean |SHAP| = 0.107, ≈30% of total importance), followed by logLDAR (0.059, 17%) (Figure 7). Both the MLP and penalized LR significantly outperformed the six traditional severity scores (AUC 0.597–0.706, all *p* < 0.001), indicating that the predictive advantage resides in the Lasso-selected feature set rather than in neural network complexity (Figures 8A–D).

**Figure 5:**
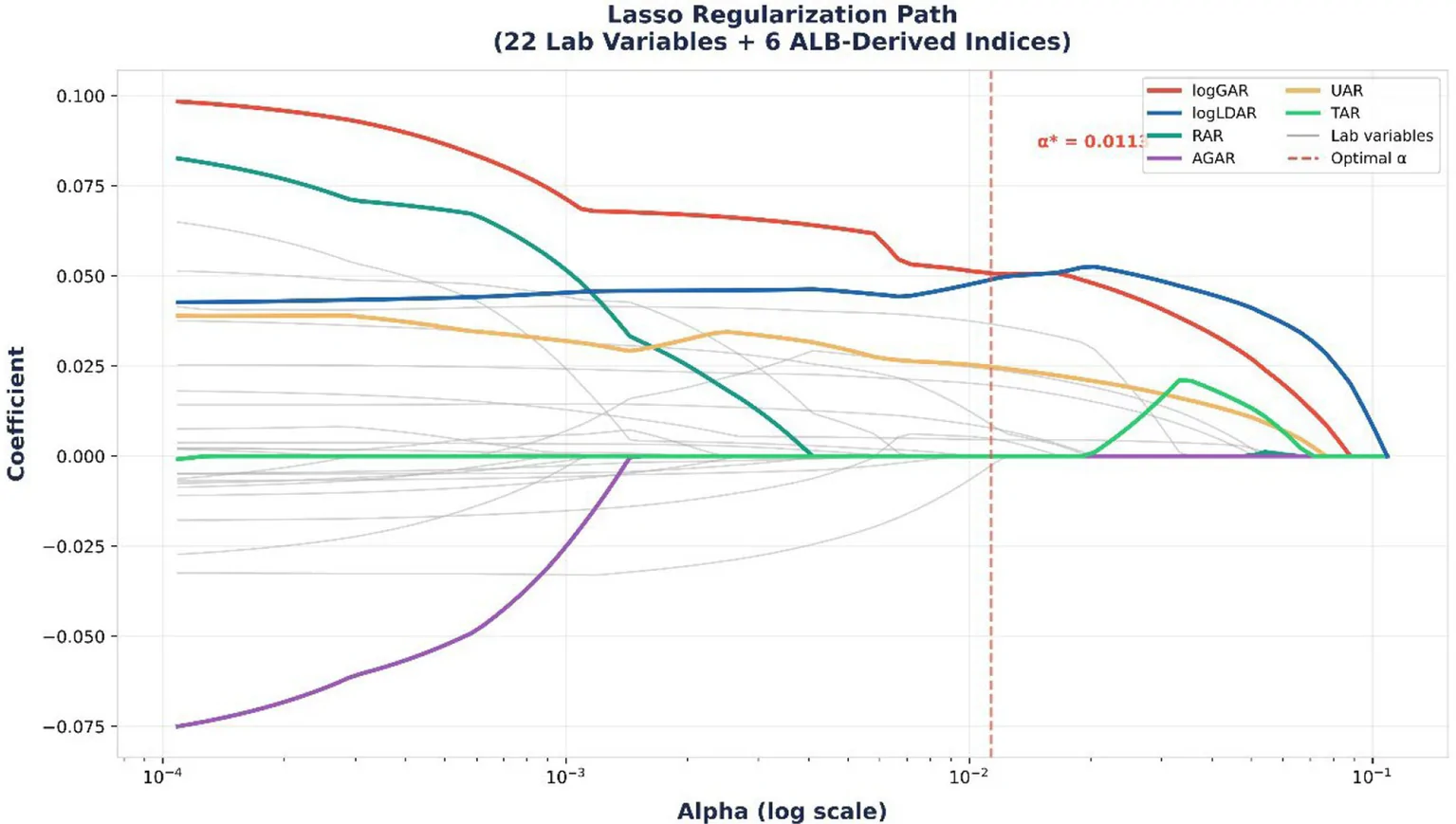
Lasso regression for feature selection. Coefficient profiles of 28 features as a function of the L1 penalty *λ* (log scale). Optimal *λ* (0.0113) selected by 10-fold cross-validation (*λ*.min, left dashed line). At this *λ*, eight features had non-zero coefficients: LogGAR, logLDAR, RDW, PT, PTT, TB, URE, and AG. All other features were shrunk to zero.

**Figure 6:**
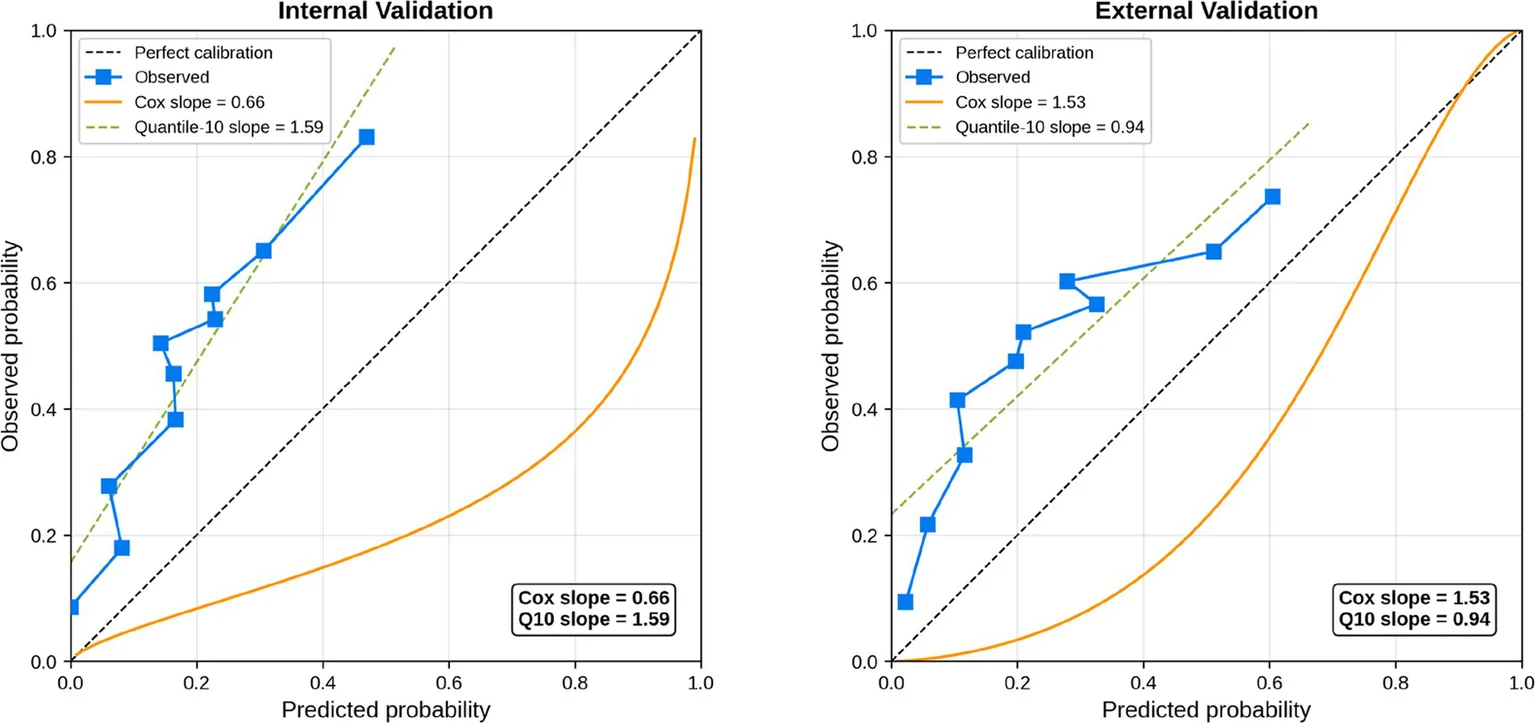
Calibration curves of the MLP model in the validation (slope 0.66) and external (slope 0.91) cohorts.

**Figure 7:**
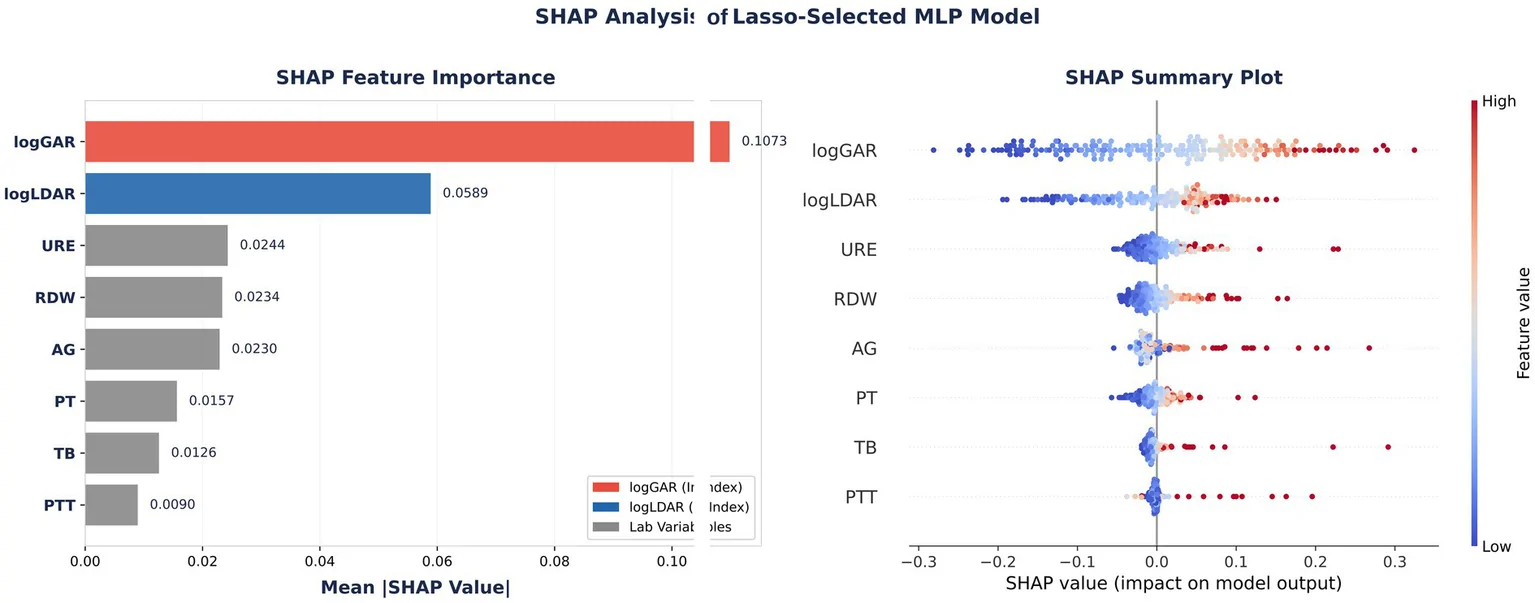
SHAP feature importance for the Lasso-MLP model. Mean |SHAP| values showing LogGAR as top predictor (0.107, ≈30% of total importance), followed by logLDAR (0.059, 17%). Features ordered by decreasing importance.

**Figure 8:**
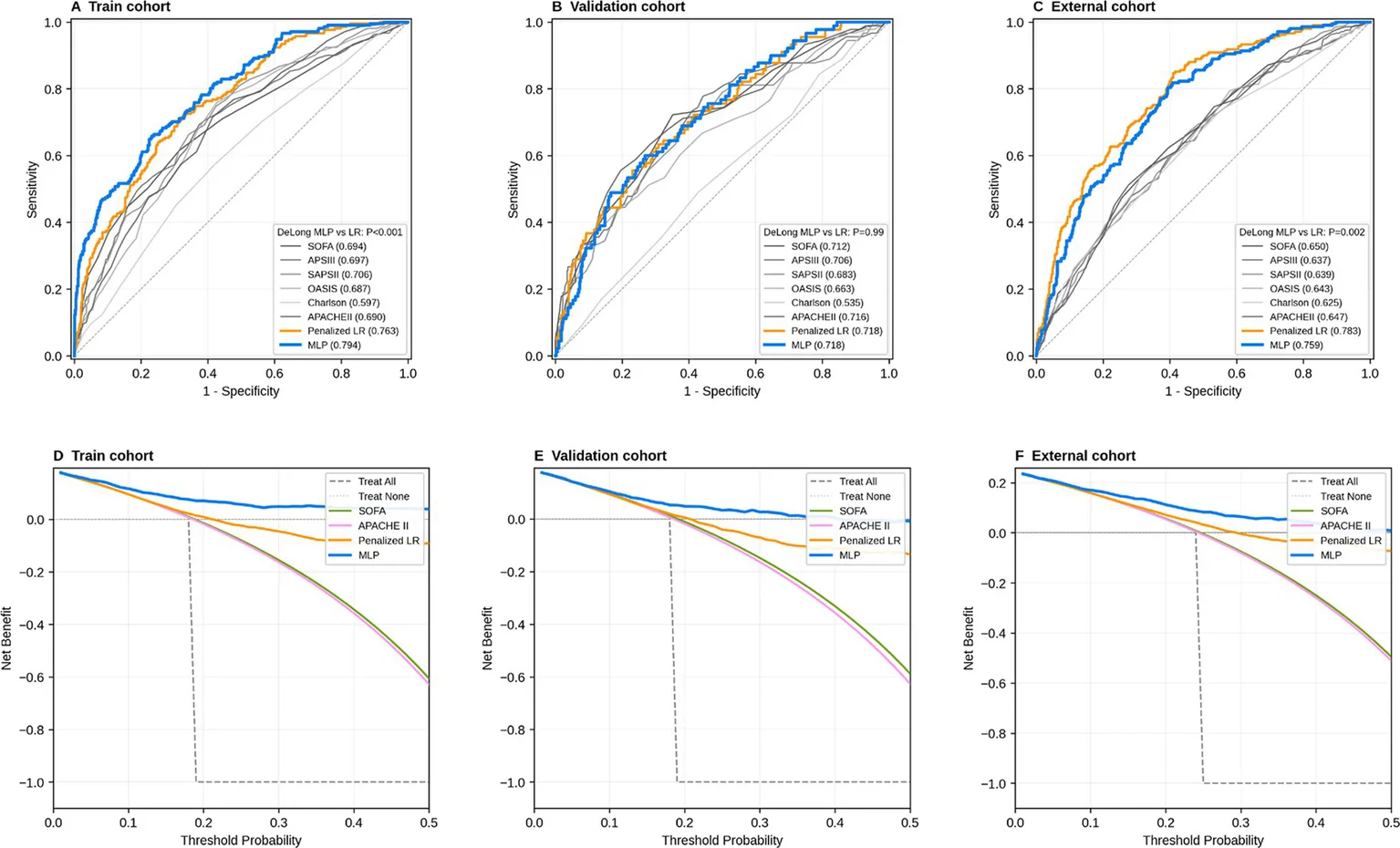
ROC and decision curve analysis of the MLP, penalized LR, and six severity scores. **(A–C)** ROC curves in the training (A), internal validation (B), and external (C) cohorts. The AUC values and DeLong test significance are shown. **(D–F)** Decision curve analysis showing net benefit across threshold probabilities (0–0.5) in the training (D), validation (E), and external (F) cohorts.

## Discussion

ICH is the most destructive stroke subtype, accounting for 10%–15% of all strokes, with a 30-day mortality exceeding 40% and most survivors facing severe disability (1–3). Despite advances in neurocritical care, prognostication remains challenging. Increasing attention has focused on nutritional status in acute ICH, as metabolic and nutritional parameters are prognostic yet largely absent from existing scores (9, 10, 29, 30). Thus, identifying easily accessible biomarkers reflecting both nutritional imbalance and metabolic stress is a research priority. To this end, we analyzed a derivation cohort of 1,625 ICH patients from MIMIC-IV and an external cohort of 861 patients from two Chinese centers, evaluating LogGAR for 28-day ICU mortality, comparing it head-to-head with five albumin-derived indices, and building Lasso-based prediction models. LogGAR was independently associated with poor prognosis, with discriminative ability comparable to logLDAR and superior to the remaining four indices, and the Lasso-selected feature set outperformed traditional severity scores.

Mechanistically, the prognostic value of LogGAR in ICH reflects its two components. Acute ICH activates the hypothalamic–pituitary–adrenal axis, releasing catecholamines, cortisol, and glucagon, which promote hepatic glycogenolysis and gluconeogenesis while inducing peripheral insulin resistance—collectively driving stress-induced hyperglycemia (SIH) (12, 13). SIH exacerbates oxidative stress, neuroinflammation, blood–brain barrier disruption, and hematoma expansion, with effects exceeding those of chronic hyperglycemia (12, 13, 31–33). Concurrently, serum albumin (ALB) maintains colloid osmotic pressure, reduces oxidative stress, and modulates inflammation (14, 15); its level reflects both nutritional status and systemic inflammatory burden (14, 15). In ICH, hypoalbuminemia and inflammation form a vicious cycle: inflammation suppresses ALB synthesis, while hypoalbuminemia impairs antioxidant and immune functions, aggravating secondary injury (34–39). Thus, GAR integrates SIH and hypoalbuminemia into a single ratio, capturing both metabolic stress and nutritional-immune reserve depletion beyond either component alone. However, without HbA1c or SHR, we could not distinguish SIH from chronic hyperglycemia, and the first glucose measurement was influenced by dextrose-containing fluids, insulin, glucocorticoids, and enteral nutrition timing, representing a pragmatic early-ICU snapshot rather than a standardized fasting measurement.

Furthermore, subgroup analysis revealed heterogeneity in the prognostic effect of LogGAR. After Benjamini–Hochberg correction for multiplicity, only the AKI interaction remained statistically significant (FDR = 0.029); the diabetes (FDR = 0.134) and COPD (FDR = 0.142) interactions were reclassified as exploratory and should be interpreted with caution. In most subgroups, including age, race, HTN, CKD, HLD and IHD, the association between LogGAR and 28-day mortality remained stable (interaction *p* > 0.05), indicating broad applicability of its prognostic value. The AKI interaction can be understood from a pathophysiological perspective: patients with AKI often have renal proteinuria and dilutional hypoalbuminemia due to fluid overload; the decrease in ALB does not mainly reflect stress-induced inflammatory depletion, but rather renal loss and hemodilution, thereby affecting the specificity of the ALB denominator in GAR (40, 41). The exploratory diabetes and COPD interactions may reflect chronic baseline hyperglycemia and chronic systemic inflammation, respectively, but require validation in independent cohorts before mechanistic interpretation.

This study systematically compared six albumin-derived composite indices within the framework of ICH prognosis assessment. The results showed that LogGAR and logLDAR exhibited comparable discriminative ability in the derivation cohort (AUC 0.679 vs. 0.681, DeLong *p* = 0.78), both superior to the remaining four indices (RAR, UAR, TAR, AGAR). In the external cohort, LogGAR achieved the highest AUC (0.691 vs. 0.664 for logLDAR). While their AUCs were comparable in the derivation cohort, LogGAR demonstrated superior performance in terms of reclassification improvement (cNRI: 0.182 vs. 0.162), effect size (HR: 1.81 vs. 1.588), and feature importance (SHAP: 0.107 vs. 0.059), suggesting that LogGAR captures a broader spectrum of the metabolic-nutritional derangements central to ICH pathophysiology.

From a mechanistic perspective, the increase in LDH reflected by logLDAR has prognostic value in diseases where systemic tissue hypoxia is the core feature, such as sepsis and acute respiratory distress syndrome (ARDS) (42), but in ICH it mainly reflects systemic secondary injury and has less specificity than glucose indices. RAR integrates information from RDW and ALB and performs well in diseases with a marked chronic inflammatory burden (43, 44), but RDW is influenced by many factors such as anemia, iron metabolism and chronic inflammation (45, 46), and its change lags behind, making it insufficiently sensitive to acute stress in ICH (44). TAR was independently significant, suggesting that the liver dysfunction or hemolytic burden reflected by elevated bilirubin independently contributes to the risk of death in ICH, possibly related to the process of multiple organ dysfunction (47). The predictive performance of AGAR and UAR was weaker because their components (AG, urea nitrogen) are easily disturbed by multiple factors such as renal function, acid–base balance and volume status, and the ratio with ALB provides limited additional information in the specific pathological dimensions of ICH (48). In summary, the competitive performance of LogGAR is determined by its ability to simultaneously capture the core pathophysiological features of ICH: “acute SIH” and “inflammatory hypoalbuminemia”.

Lasso regression selected eight core features from 28 candidate variables, with LogGAR and logLDAR ranking as the top two contributors. Critically, a penalized logistic regression on the identical eight features achieved performance comparable to the MLP (validation AUC 0.718 for both; external AUC 0.783 vs. 0.759), indicating that the network’s nonlinearity did not add predictive value on these tabular predictors. This suggests that the predictive signal lies primarily in the feature set rather than model complexity—favoring interpretable linear models when nonlinearity provides no added benefit. Of note, the six conventional severity scores were used as fixed published instruments and were not refit on our data, which may inflate the apparent advantage of both models.

Calibration analysis showed suboptimal calibration in the internal validation set (slope 0.66), likely reflecting overfitting, which may be attributable in part to the relatively small validation sample (*n* = 488) that renders calibration estimates less stable. Calibration improved substantially in the external cohort (slope 0.91), approaching the ideal value. Decision curve analysis demonstrated that both models provided higher net benefit than conventional severity scores across clinically relevant thresholds. SHAP analysis confirmed LogGAR as the most important predictor. While the “black box” nature of MLP limits clinical translation, the comparable performance of the transparent penalized logistic regression offers an interpretable alternative with equivalent discriminative ability.

Previous studies have validated GAR as a prognostic biomarker in ICH (16). Building on this foundation, the present study contributes three key novelties. First, we provide the first systematic head-to-head comparison of GAR with five other albumin-derived indices—logLDAR, RAR, UAR, TAR, and AGAR—in the ICH population, clarifying its relative discriminative performance across multiple dimensions (AUC, reclassification, and feature importance). Second, we focus on the 28-day ICU mortality endpoint, a clinically actionable timeframe for acute intervention, distinct from the 90-day outcomes reported previously. Third, we compare an MLP against a penalized logistic regression on identical Lasso-selected features, demonstrating that nonlinearity does not add predictive value—a finding that favors simpler, interpretable models for ICU prognostic research. Collectively, these contributions position our study as a meaningful extension rather than a replication of prior work.

Several limitations should be acknowledged. First, the retrospective design can only demonstrate association, not causation; whether interventions targeting LogGAR improve outcomes remains untested. Second, the MIMIC-IV derivation cohort did not include ICH-specific imaging variables in the primary data extraction. While these factors are well-established prognostic indicators, we addressed this through two complementary approaches: a sensitivity analysis incorporating GCS into Model 3 yielded virtually identical results to the primary analysis (HR = 1.79 vs. 1.81), and in the external cohort—where hematoma volume, location, IVH, expansion, and neurosurgical intervention were fully available and adjusted for—LogGAR remained independently associated with mortality (HR = 3.77, *p* < 0.001). These analyses collectively indicate that the absence of these variables in the derivation cohort does not materially affect our conclusions. Third, LogGAR was derived from a single initial glucose measurement, which may be influenced by dextrose-containing fluids, insulin, or glucocorticoids, and without HbA1c or stress hyperglycemia ratio, we could not differentiate stress-induced from chronic hyperglycemia.

Fourth, the external validation cohort originated from two Chinese centers, and the moderate AUC values (0.69–0.78) suggest cautious interpretation for clinical implementation. Fifth, the 28-day ICU mortality endpoint does not capture post-ICU discharge deaths before day 28; however, the Fine–Gray competing-risk analysis treating ICU discharge as a competing event yielded consistent results (sHR = 2.01, *p* < 0.001), supporting the robustness of our findings. Sixth, the MLP and penalized LR were benchmarked against fixed severity scores rather than scores refit on our data, which may inflate their apparent advantage; additionally, the external cohort had different case-mix (24.3% vs. 18.5% mortality), reflecting healthcare system variations.

## Conclusion

This multicenter retrospective study demonstrates that LogGAR is independently associated with 28-day ICU mortality in critically ill patients with ICH, with robustness confirmed across sensitivity analyses (I62.9 exclusion, GCS adjustment, and Fine–Gray competing-risk model). Among six albumin-derived indices, LogGAR showed AUC comparable to logLDAR but superior performance in reclassification improvement, effect size, and feature importance. The Lasso-selected eight-feature set outperformed traditional severity scores, while a penalized logistic regression matched the MLP on identical features, indicating that the predictive signal lies in the feature set rather than model complexity. As a simple, inexpensive, and readily available biomarker, LogGAR may serve as an adjunctive screening tool for early ICU risk stratification. However, moderate discriminative performance (AUC 0.69–0.78) cautions against standalone use. Whether GAR-guided interventions improve outcomes requires prospective validation. Future studies with longitudinal GAR measurements, hyperglycemia subtyping (SHR/HbA1c), and diverse populations are needed to confirm generalizability and explore causality.

## Data Availability

The original contributions presented in the study are included in the article/Supplementary material, further inquiries can be directed to the corresponding authors.
