# Supplementary material for: Head-to-head comparison of glucose-to-albumin ratio against five albumin-derived indices for predicting 28-day ICU mortality in critically ill patients with spontaneous intracerebral hemorrhage: a multicenter retrospective study
Source: Front Nutr. 2026 Oct 8;13:1909009. doi: 10.3389/fnut.2026.1909009 (PMC13687388; doi:10.3389/fnut.2026.1909009)
Supplement: Supplementary file 1 [file Table_1.DOCX]

**TRIPOD+AI Checklist**

*Transparent Reporting of a multivariable prediction model for Individual Prognosis Or Diagnosis with AI extensions*

Collins GS, Moons KGM, Dhiman P, et al. TRIPOD+AI statement: updated guidance for reporting clinical prediction models that use regression or machine learning methods. BMJ. 2024;385:e078378.

*Instructions: This checklist applies to the manuscript "Glucose-to-albumin ratio and 28-day ICU mortality in severe spontaneous intracerebral hemorrhage: a retrospective study with multicenter external validation and multi-index comparison." D = Development, E = Evaluation (external validation). Page/section references correspond to the revised manuscript (Manuscript_revised_v2.docx).*

| **Item** | **D/E** | **Section/Topic** | **Checklist Item** | **Reported / Location** |
| --- | --- | --- | --- | --- |
| 1 | D;E | Title | Identify the study as developing or evaluating the performance of a multivariable prediction model, the target population, and the outcome to be predicted. | Yes. Title page — identifies as retrospective study with multicenter external validation; target population: severe spontaneous ICH; outcome: 28-day ICU mortality |
| 2 | D;E | Abstract | See TRIPOD+AI for Abstracts checklist. | Yes. Abstract — structured abstract with background, methods, results, conclusion |
| 3a | D;E | Background | Explain the healthcare context (including whether diagnostic or prognostic) and rationale for developing or evaluating the prediction model, including references to existing models. | Yes. Introduction — prognostic context; references to He et al. 2023 [48] and existing scoring systems [6-8] |
| 3b | D;E | Background | Describe the target population and the intended purpose of the prediction model in the context of the care pathway, including its intended users. | Yes. Introduction — target population: critically ill ICH patients in ICU; intended users: neurocritical care clinicians |
| 3c | D;E | Background | Describe any known health inequalities between sociodemographic groups. | Partial. Not explicitly discussed; acknowledged as limitation (external validity across ethnicities/healthcare systems) |
| 4 | D;E | Objectives | Specify the study objectives, including whether the study describes the development or validation of a prediction model (or both). | Yes. Introduction (last paragraph) — both development (MIMIC-IV) and external validation (two-center cohort) |
| 5a | D;E | Data | Describe the sources of data separately for the development and evaluation datasets, the rationale for using these data, and representativeness of the data. | Yes. Methods, Data sources — MIMIC-IV (v2.2) for derivation; PLA General Hospital (n=321) + Guizhou Medical University (n=540) for external validation |
| 5b | D;E | Data | Specify the dates of the collected participant data, including start and end of participant accrual; and, if applicable, end of follow-up. | Yes. Methods — MIMIC-IV: 2008-2019; External: January 2025 to June 2026; Follow-up: 28 days |
| 6a | D;E | Participants | Specify key elements of the study setting including the number and location of centres. | Yes. Methods — derivation: BIDMC ICU (MIMIC-IV); external: two centers (Beijing PLA General Hospital, Guizhou Medical University) |
| 6b | D;E | Participants | Describe the eligibility criteria for study participants. | Yes. Methods, Study population — inclusion: age >=18, ICD-9 431 or ICD-10 I610-I619/I62.9, ICU admission; exclusion: death <24h, multiple admissions, missing labs |
| 6c | D;E | Participants | Give details of any treatments received, and how they were handled during model development or evaluation, if relevant. | Yes. Methods — ventilation, CRRT, vasopressors, glucocorticoids, antihypertensives, antibiotics, sedation included as covariates; neurosurgery in external cohort |
| 7 | D;E | Data preparation | Describe any data pre-processing and quality checking, including whether this was similar across relevant sociodemographic groups. | Yes. Methods, Variable extraction — PostgreSQL extraction; missing data handled via multiple imputation (mice, m=10, maxit=50); unit harmonization across cohorts |
| 8a | D;E | Outcome | Clearly define the outcome that is being predicted and the time horizon, including how and when assessed. | Yes. Methods — 28-day ICU mortality; defined as death in ICU within 28 days of admission; patients discharged alive before day 28 censored at discharge |
| 8b | D;E | Outcome | If outcome assessment requires subjective interpretation, describe the qualifications and demographic characteristics of the outcome assessors. | Yes. Outcome is mortality (objective); no subjective interpretation required |
| 8c | D;E | Outcome | Report any actions to blind assessment of the outcome to be predicted. | Yes. Outcome is mortality (objective); blinding not applicable |
| 9a | D | Predictors | Describe the choice of initial predictors and any pre-selection of predictors before model building. | Yes. Methods — 28 candidate features: 22 lab variables with significant univariate associations + 6 albumin-derived indices; Lasso for selection |
| 9b | D;E | Predictors | Clearly define all predictors, including how and when they were measured. | Yes. Methods, Variable extraction — six domains defined; GAR = Glu/ALB; first ICU measurement; log-transformed |
| 9c | D;E | Predictors | If predictor measurement requires subjective interpretation, describe the qualifications and demographic characteristics of the predictor assessors. | Yes. Predictors are laboratory values (objective); no subjective interpretation required |
| 10 | D;E | Sample size | Explain how the study size was arrived at, and justify that the study size was sufficient. | Partial. No formal sample size calculation; all eligible patients included; events-per-variable ratio reported (41.8 for external parsimonious model) |
| 11 | D;E | Missing data | Describe how missing data were handled. Provide reasons for omitting any data. | Yes. Methods — multiple imputation (mice v3.16, m=10, maxit=50, PMM); 19/40 variables had 1.1-5.9% missingness; variables >30% excluded; convergence confirmed (PSRF <1.05); complete-case sensitivity analysis performed |
| 12a | D | Analytical methods | Describe how the data were used in the analysis, including whether the data were partitioned. | Yes. Methods — 70/30 stratified split (seed=42); training n=1,137, validation n=488; external validation n=861 |
| 12b | D | Analytical methods | Describe how predictors were handled in the analyses (functional form, rescaling, transformation, standardisation). | Yes. Methods — LogGAR (natural log-transformed); z-score normalization fitted on training set; RCS with knots at 5th/35th/65th/95th percentiles |
| 12c | D | Analytical methods | Specify the type of model, rationale, all model-building steps, including hyperparameter tuning, and method for internal validation. | Yes. Methods — MLP (3 hidden layers 128/64/32, batch norm, ReLU, dropout 0.3); Adam (lr=0.001, weight_decay=1e-4); ReduceLROnPlateau; early stopping (patience=20); Lasso (10-fold CV, lambda=0.0113); penalized logistic regression (L2 ridge) as benchmark |
| 12d | D;E | Analytical methods | Describe if and how any heterogeneity in estimates of model parameter values and model performance was handled across clusters. | Partial. Two external centers not analyzed separately; acknowledged as limitation; cross-cohort comparison table provided |
| 12e | D;E | Analytical methods | Specify all measures and plots used to evaluate model performance (discrimination, calibration, clinical utility) and to compare multiple models. | Yes. Methods — AUC with DeLong test; cNRI + continuous NRI + IDI; calibration plots (slope/intercept); DCA; SHAP; Brier score; AUPRC; RCS; KM curves; Fine-Gray competing risks |
| 12f | E | Analytical methods | Describe any model updating arising from the model evaluation. | Yes. No model updating performed; model applied as-is to external cohort |
| 12g | E | Analytical methods | For model evaluation, describe how the model predictions were calculated. | Yes. Methods — MLP trained on derivation training set; predictions generated on external cohort using same preprocessing pipeline |
| 13 | D;E | Class imbalance | If class imbalance methods were used, state why and how this was done, and any subsequent methods to recalibrate the model. | Yes. Methods — weighted BCE loss (pos_weight=4.39) for event rate 18.5%; calibration assessed post-hoc (slope 0.33 validation, 0.91 external) |
| 14 | D;E | Fairness | Describe any approaches that were used to address model fairness and their rationale. | Partial. Subgroup analyses by age, sex, race, comorbidities performed; no formal fairness metrics (e.g., equalized odds) computed; acknowledged as limitation |
| 15 | D | Model output | Specify the output of the prediction model. Provide details and rationale for any classification and how thresholds were identified. | Yes. Methods — output: predicted probability of 28-day ICU mortality; Youden index for optimal cutoff (3.638); risk categories <10%, 10-20%, >20% for cNRI |
| 16 | D;E | Development vs evaluation | Identify any differences between the development and evaluation data in healthcare setting, eligibility criteria, outcome, and predictors. | Yes. Results & Discussion — external cohort younger (58 vs 67), more severe (SOFA 6 vs 4), higher mortality (24.3% vs 18.5%); external has GCS/hematoma variables not in derivation; cross-cohort comparison table provided |
| 17 | D;E | Ethics | Name the institutional research board or ethics committee that approved the study and describe informed consent procedures. | Yes. Methods — MIT IRB for MIMIC-IV; Ethics Committee of Affiliated Hospital of Guizhou Medical University (approval No. 2025-0432) and Ethics Committee of Chinese PLA General Hospital, both waived informed consent. |
| 18 | D;E | Patient involvement | Describe the involvement of patients or members of the public in the design, conduct, reporting, or dissemination of the study or state no involvement. | Yes. No patient or public involvement; stated in cover letter |
| 19a | D;E | Reproducibility | Describe the availability of the study data. | Yes. MIMIC-IV is publicly available; external data available on reasonable request with appropriate ethics approval |
| 19b | D;E | Reproducibility | Describe the availability of the study code. | Partial. R and Python code described in Methods; full code availability to be confirmed upon publication |
| 20a | D;E | Participants (Results) | Describe the flow of participants through the study. A diagram may be helpful. | Yes. Supplementary Figure S2 (CONSORT-style flowchart) showing MIMIC-IV derivation cohort selection with exclusion criteria and counts; Table 1 and Table S1 for baseline characteristics. |
| 20b | D;E | Participants (Results) | Report the characteristics overall and for each data source, including key dates, predictors, treatments, sample size, outcome events, follow-up time, and missing data. | Yes. Table 1 (derivation by GAR tertiles); Table S1 (derivation by mortality); Table S2 (external by mortality); cross-cohort comparison table added |
| 20c | E | Participants (Results) | For model evaluation, show a comparison with the development data of the distribution of important predictors. | Yes. Cross-cohort comparison table with SMD for all key variables; case-mix differences discussed in Results and Discussion |
| 21 | D;E | Model development | Specify the number of participants and outcome events in each analysis. | Yes. Results — derivation: 1,625 (301 events); training: 1,137 (211 events); validation: 488 (90 events); external: 861 (209 events) |
| 22 | D | Model specification | Provide details of the full prediction model to allow predictions in new individuals. | Partial. Lasso coefficients reported; MLP architecture described; penalized LR described; full model weights/code to be provided as supplementary upon publication |
| 23a | D;E | Model performance | Report model performance estimates with confidence intervals, including for key subgroups. Consider plots. | Yes. Results — AUC with 95% CI; HR with 95% CI; calibration slopes; DCA; forest plot for subgroups; Figures 1-8 |
| 23b | D;E | Model performance | If examined, report results of any heterogeneity in model performance across clusters. | Partial. Subgroup analyses performed (Figure 3); two external centers not analyzed separately; acknowledged as limitation |
| 24 | E | Model updating | Report the results from any model updating. | Yes. No model updating performed |
| 25 | D;E | Interpretation | Give an overall interpretation of the main results, including issues of fairness in the context of the objectives and previous studies. | Yes. Discussion — results interpreted in context of He et al. 2023; SIH mechanism discussed; subgroup heterogeneity (AKI) discussed; fairness across demographic groups addressed via subgroup analyses |
| 26 | D;E | Limitations | Discuss any limitations of the study and their effects on biases, statistical uncertainty, and generalizability. | Yes. Discussion — 11 limitations explicitly listed: retrospective design, ICU-only population, two Chinese centers, missing GCS/hematoma in derivation, single glucose measurement, I62.9 misclassification, moderate AUC, fixed severity scores, MAR assumption, outcome definition, lack of HbA1c/SHR |
| 27a | D | Usability | Describe how poor quality or unavailable input data should be assessed and handled when implementing the prediction model. | Partial. Missing data handling described (imputation); implementation guidance for missing predictor values not explicitly discussed |
| 27b | D;E | Usability | Discuss any next steps for future research, with a specific view to applicability and generalizability. | Yes. Discussion & Conclusion — prospective interventional studies needed; longitudinal GAR measurements; multicentre studies with diverse populations; HbA1c/SHR sensitivity analyses; 28-day all-cause mortality sensitivity analysis |

**Summary: Of 27 checklist items (with sub-items totaling 47 entries), 39 are fully reported (Yes), 8 are partially reported (Partial), and 0 are missing (No). All partially reported items are acknowledged as limitations in the revised manuscript.**
